# Comparison of widefield swept-source optical coherence tomographic angiography and fluorescein fundus angiography for detection of retinal neovascularization with diabetic retinopathy

**DOI:** 10.1186/s12886-023-03073-2

**Published:** 2023-07-12

**Authors:** Yanyan Yang, Fengjiao Li, Tingting Liu, Wanzhen Jiao, Bojun Zhao

**Affiliations:** 1 Department of Ophthalmology, Shandong Provincial Hospital, Shandong University, Jinan, 250021 Shandong China; 2 Department of Ophthalmology, Liaocheng People’s Hospital, Liaocheng, 252000 Shandong China; 3 Department of Ophthalmology, Shandong Provincial Hospital Affiliated to Shandong First Medical University, Jinan, China; 4 Eye Hospital of Shandong First Medical University (Shandong Eye Hospital), Jinan, China; 5 State Key Laboratory Cultivation Base, Shandong Provincial Key Laboratory of Ophthalmology, Shandong Eye Institute, Shandong First Medical University & Shandong Academy of Medical Sciences, Qingdao, China; 6 School of Ophthalmology, Shandong First Medical University, Jinan, China

**Keywords:** Fluorescein fundus angiography, Neovascularization, Proliferative diabetic retinopathy, Vitreous angiomosaic image, Widefield swept-source optical coherence tomographic angiography

## Abstract

**Background:**

To compare vitreous angiomosaic images (VAMIs), obtained by widefield swept-source optical coherence tomographic angiography (wfSS-OCTA) and the image of fluorescein fundus angiography (FFA) in the identification of retinal neovascularization (NV) in patients with diabetic retinopathy (DR).

**Methods:**

In this prospective observational study, severe non-proliferative diabetic retinopathy (NPDR) or proliferative DR (PDR) patients were included. All patients underwent FFA and wfSS-OCTA. The number of NVs identified by wfSS-OCTA VAMIs using five fixations 12 × 12 mm montage scans and the resembling FFA images were compared.

**Results:**

Fifty-three eyes of 29 patients were enrolled. NVs were detected in 25 eyes by using FFA, including 9 NVs of the disc (NVDs) and 72 NVs elsewhere (NVEs), and in 29 eyes by OCTA, including 11 NVDs and 90 NVEs. The detection rate of NV and NVD of OCTA was comparable to that of FFA (p > 0.05), and the level of agreement was excellent (κ = 0.850, κ = 0.754). Using FFA as the gold standard, the sensitivity for detection of NV by OCTA was 100.0%, specificity was 85.7%, the positive-predictive value was 86.2%, and the negative-predictive value was 100.0%. Compared with FFA, OCTA was superior in terms of the number of NVEs identified (p = 0.024). When we excluded images of patients treated with anti-vascular endothelial growth factor (VEGF) intravitreal therapy for < 3 months, OCTA was comparable to FFA in terms of the number of NVEs discovered (*p* = 0.203), with excellent agreement (intraclass correlation coefficient = 0.941).

**Conclusions:**

WfSS-OCTA is an independent non-invasive alternative to FFA for NV discovery, NVD detection, and individual NVE identification, particularly in patients with PDR who have a history of prior treatment with anti-VEGF.

## Background

Diabetic retinopathy (DR) is the leading cause of blindness among the global working-age population [1–3]. Proliferative DR (PDR) is an advanced stage of DR characterized by the appearance of neovascularization (NV) [4, 5]. If not detected and treated during the early stage, the NV vessels rupture, leading to vitreous hemorrhage (VH) and retinal detachment, which cause blindness [6]. In more severe cases, the NV grows on the anterior chamber angle structures, leading to neovascular glaucoma (NVG). Therefore, early detection is essential for NV control, which may prevent vision loss in patients with DR [7].

Fluorescein fundus angiography (FFA) has been used for identifying NV for over 50 years [8–11]; however, several limitations restrict its clinical application. First, it is an invasive procedure accompanied by dye-related side effects such as nausea, anaphylaxis, and even death. Therefore, FFA is unsuitable for patients with diabetic renal disorders and severe hypertension, and for pregnant patients, who have a higher risk of DR progression. Second, FFA images are two-dimensional (2D), which makes it difficult to distinguish whether the abnormal blood vessels with moderate dye leakage are intraretinal microvascular abnormalities (IRMA) or NV. Third, in the late stage of FFA, dye leakage due to IRMA, NV, microaneurysms (MAs), or other conditions involving increased permeability causes blurred images that obscure details of the abnormal vessels. Therefore, more advanced examinations are needed for detection of NV and for diagnosis of PDR.

In contrast, optical coherence tomography angiography (OCTA) is a non-invasive and safe procedure, providing clear three-dimensional (3D) images of blood vessels and structures without the need to use dye agents. Generally, OCTA is used to diagnose macular or optic disc diseases and as an alternative to FFA [12, 13]. Widefield swept-source optical coherence tomographic angiography (wfSS-OCTA) has been used to distinguish IRMA from NV [14]. NVs in the retina and optic nerve protruding into the vitreous cavity and showing significant leakage on FFA are diagnostic hallmarks of PDR [15, 16]. Vitreoretinal interface (VRI) slab images showing hyper-reflected blood flow from the retinal surface to the vitreous cavity was proven to be suitable for identification of NV in patients with PDR [16–21]. As it is restricted by the narrow scan area (3 × 3 mm or 6 × 6 mm), OCTA cannot be used independently to differentiate PDR from DR [22]. Larger scan areas, however, result in decreased resolution of the fine retinal vessels. However, a montage approach can be used to scan a larger field while maintaining the high resolution of the vasculature [23]. Recently, a retrospective study reported that the detection rate of PDR with wfSS-OCTA using 12 × 12 mm montage scan was higher than that achieved by clinical examination [20].

WfSS-OCTA using 15 × 15 mm montage scan plus ultra-widefield color fundus photography may offer a less invasive alternative to FFA for DR diagnosis [9]. WfSS-OCTA can auto-merge 12 × 12 mm fundus scans obtained at five fixation points into different slabs of an angiomosaic image (AMI), with a scan area representing up to 17.5 × 23.5 mm of the retina surface. The OCTA vitreous AMI (VAMI) shows the hyper-reflected flow from the retinal surface to the vitreous cavity, which can be used to diagnose retinal NV in patients with PDR.

Here, we conducted a prospective study to compare five-fixations of 12 × 12 mm scan montage VAMI up to 17.5 × 23.5 mm of the retina surface obtained by wfSS-OCTA with resembling FFA images for the detection of NV.

## Methods

### Subjects

This prospective observational study was approved by the ethics committee of Shandong Provincial Hospital (approval number 2021-058) and was performed in accordance with the principles of the Declaration of Helsinki. Informed consent was obtained from all patients. Severe non-proliferative DR (NPDR) or PDR patients with type 2 diabetes mellitus (DM) based on clinical diagnosis were consecutively included in this study at Shandong Provincial Hospital from September 2020 to January 2021. The exclusion criteria were as follows: patients with severe media opacities (such as cataracts, corneal edema, and diffuse VH), fixation difficulty, acute angle-closure glaucoma, concomitant chorioretinal diseases, unavailable FFA images, unavailable automatic image montage, poor image quality, and a history of vitrectomy. All enrolled patients underwent routine ophthalmologic examination, FFA (Heidelberg Spectralis HRA; Heidelberg Engineering, Heidelberg, Germany), and wfSS-OCTA (VG200, SVision Imaging, Ltd., Luoyang, China). Clinical information, including diabetes duration and DR severity, was collected from the medical records.

### Image acquisition and analysis

WfSS- OCTA uses a swept-source laser with a central wavelength of 1,050 nm and a bandwidth of 100 nm at a scanning speed of 200,000 A per second. It provides 3D images of vessels and structures with an axial resolution of 5μm, digital resolution of 2.0μm, and scanning depth of 2.7 mm. This study used the 12×12 mm 1024×1024 R2 angio scan protocol in five prediction fixations (including central, superotemporal, inferotemporal, inferonasal, and superonasal areas) for each patient. Each scan was comprised of 1024 B-scans with 1024 A-scans per B-scan, repeated twice at each fixation. The 12×12 mm scans of the fundus obtained at five fixation points were auto-merged into different slabs of widefield AMI, with a scan area of up to 60°×80 (17.5 × 23.5 mm).

**Fig. 1:**
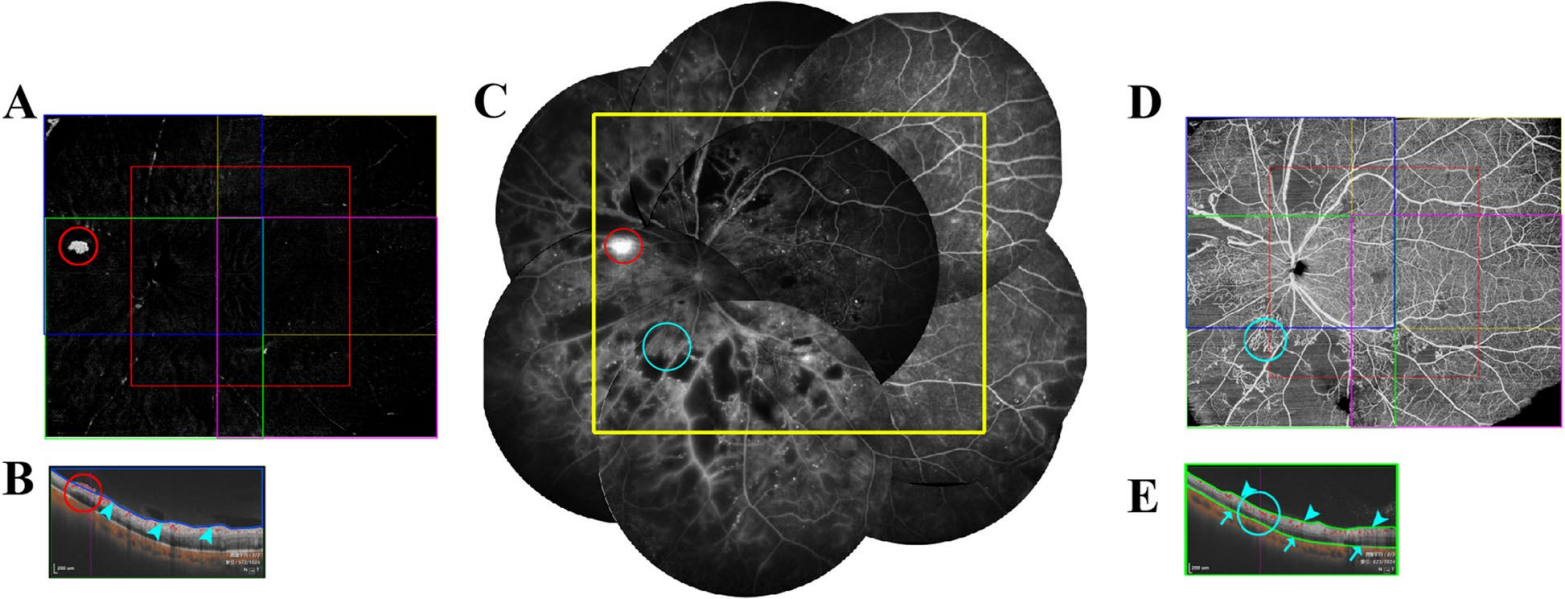
A 56-year-old man with DM for 15 years was treatment-naïve. **A** VAMI: Five fixations of vitreous enface angio-image (including central, superotemporal, inferotemporal, inferonasal, and superonasal areas) were auto-merged into VAMI. NV (red circle) was identified as abnormal vessels blood flow on wfSS-OCTA VAMI. **B** Superonasal B-scan: A 12 × 12 mm B-scan focused on the superonasal area. NV (red circle) appears as hyper-reflected blood flow, breaking the ILM into the vitreous in the B-scan. **C** Merged FFA image: The merged FFA image was cropped to create the resembling FFA image (yellow rectangle), NV (red circle) was characterized by abnormal blood vessels with definite fluorescein dye leakage, and IRMA (blue circle) was characterized by no or minimal fluorescein dye leakage. **D** RAMI: Five fixations of retinal enface angio-image (including central, superotemporal, inferotemporal, inferonasal, and superonasal areas) were auto-merged into RAMI. IRMA was identified by RAMI (blue circle). **E** Inferonasal B-scan: A 12 × 12 mm B-scan focused on the inferonasal area. IRMA (blue circle) appears as hyper-reflected blood flow within the retina. DM, diabetes mellitus; VAMI, vitreous angiomosaic image; wfSS-OCTA, widefield swept-source optical coherence tomography angiography; NV, neovascularization; ILM, internal limiting membrane; RAMI, retinal angiomosaic image; IRMA, intraretinal microvascular abnormalities; FFA, fluorescein fundus angiography

**Fig. 2:**
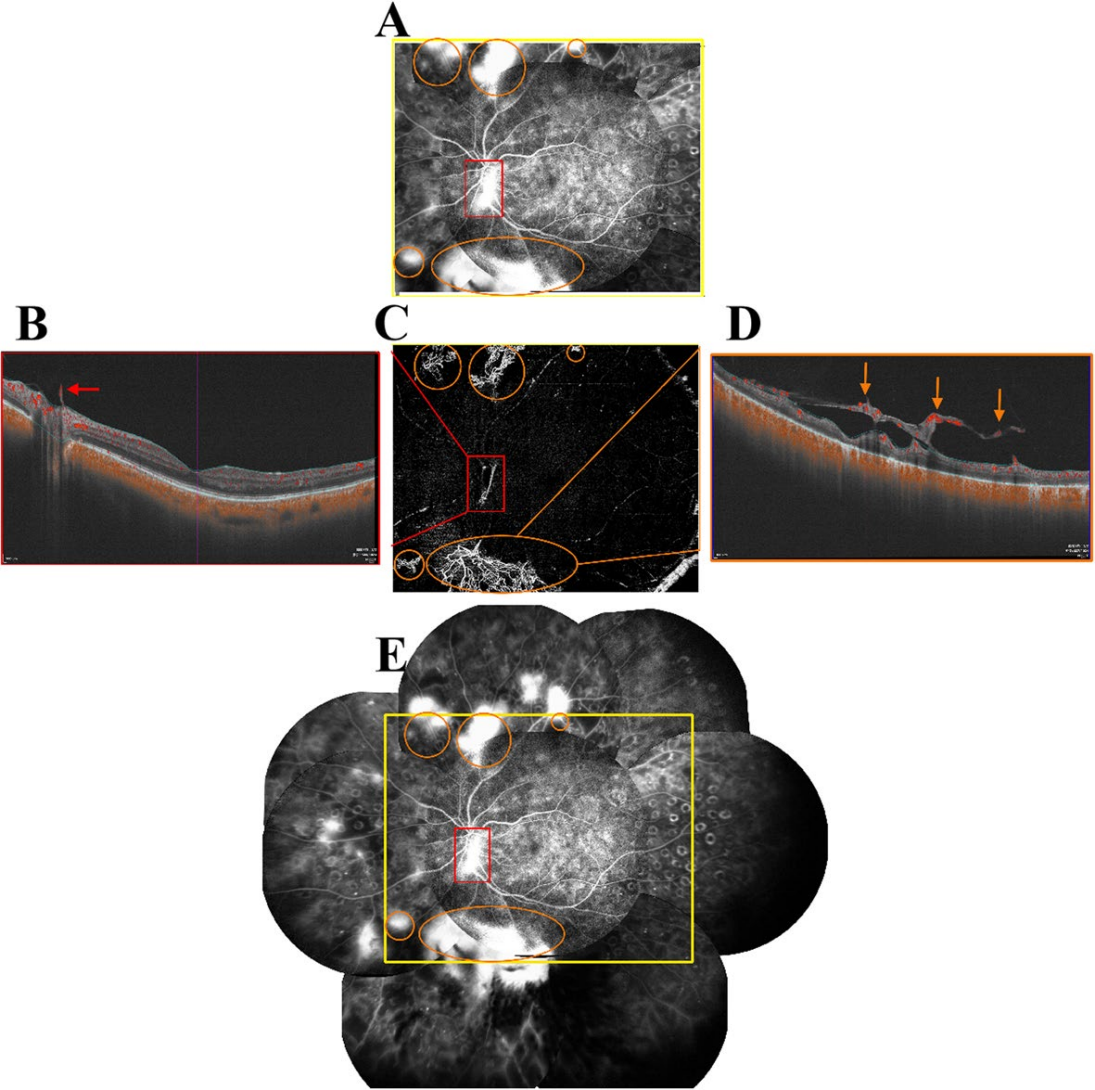
A 53-year-old woman who had DM for 10 years, with a history of panretinal photocoagulation for 8 months. **A** Resembling FFA image demonstrating multiple leakage NVEs (orange circles) and NVDs (red boxes). **B** Central B-scan: NVD (red arrow) was defined as NV located in the disc or within a 1-disc diameter from the margin. The NVD above the optic disc (red box) on FFA and VAMI was confirmed by OCT B-scan, which showed that the lesion had broken through the ILM. **C** VAMI: NVEs are highlighted with orange circles, NVD is shown as red box, which corresponds to the images on FFA. **D** Inferonasal B-scan: NVE (orange arrow) was defined as NV located outside the area of NVD. The NVE on VAMI and FFA (orange circles) was confirmed by OCT B-scan that the lesions had broken through the ILM. **E** Merged FFA images: Majority of the NVs were inside the resembling FFA (yellow rectangle) image. DM, diabetes mellitus; FFA, fluorescein fundus angiography; NVD, neovascularization of the disc; NVE, neovascularization elsewhere; NV, neovascularization; OCT, optical coherence tomography; VAMI, vitreous angiomosaic image; ILM, internal limiting membrane

The VAMI showed the hyper-reflected blood flow within a defined volume between the outer boundary 5μm anterior to the internal limiting membrane (ILM) and the inner boundary at the top of the vitreous cavity in the B-scan, and was the main image used to detect NV (Fig. 1A, B). NV was identified as abnormal vessels on wfSS-OCTA VAMI (Fig. 1A), including NV of the disc (NVD) and NV elsewhere (NVE). NVD was defined as NV located in the disc or within a 1-disc diameter from the margin (Fig. 2B, C). NVE was defined as NV located outside this area (Fig. 2C, D). The retinal AMI (RAMI) showed the hyper-reflected signals within a defined volume between the outer boundary 10μm above the Bruch’s membrane and the inner boundary 5μm above the ILM and was the main image used to differentiate IRMA from NV (Fig. 1D, E). We detected IRMA as dilated, tortuous, and looped abnormal vessels adjacent to areas of capillary loss on wfSS-OCTA RAMI (Fig. 1D). All defined boundaries were automatically segmented using the wfSS-OCTA device. Each B-scan was used to confirm the diagnosis and manual segmentation were carried out in cases of segmentation error.

FFA was performed as a standard protocol after an intravenous injection of 5 mL of 10% sodium fluorescein into an antecubital vein. Images were captured digitally and compressed into high-quality JPEG files. For evaluation, the FFA images were transferred to Adobe Photoshop software (Adobe System, Inc, San Jose, CA), spliced according to the direction of the blood vessels, and cropped to the same location and size as that on wfSS-OCTA RAMI, which was labelled as “resembling FFA image” (Figs. 1C, 2A, E). On FFA, NV was characterized by abnormal blood vessels with definite fluorescein dye leakage, and IRMA was characterized by no or minimal fluorescein dye leakage (Fig. 1C). The FFA images and wfSS-OCTA VAMIs were read by two masked graders (YYY and LFJ) independently at different time points in random order. Image evaluation was performed in a standardized, dim environment on the instrument display screen. A third senior ophthalmologist (ZBJ) adjudicated in case of disagreement between the graders. An eye with detected NV was accounted for as “1” eye with NV. An eye without NV was accounted for as “0” eye with NV. An eye detected NVD was accounted for as “1” eye with NVD, while an eye without NVD was accounted for as “0” eye with NVD. The number of NVEs were accounted separately for abnormal vessels without connection, and with at least a half disc diameter interval (Fig. 2E). NVs, NVDs, or NVEs on wfSS-OCTA VAMIs and resembling FFA images were compared.

### Statistical analysis

The normally distributed continuous variables are presented as mean ± standard deviations. The detection rate of NV/NVD was compared between wfSS-OCTA VAMIs and resembling FFA images by means of McNemar’s test. Levels of agreement were calculated using Cohen’s kappa test. Kappa was characterized as follows: > 0.75 as excellent, 0.40–0.75 as fair to good, and < 0.40 as poor. Resembling FFA images were used as the gold standard. The sensitivity, specificity, and accuracy for discovering NV with wfSS-OCTA VAMI were calculated. The number of NVEs identified on wfSS-OCTA VAMIs and on FFA images were compared using the nonparametric Wilcoxon signed-rank test. Levels of agreement were calculated using the intraclass correlation coefficient (ICC). The ICC was characterized as follows: > 0.75 as excellent, 0.40–0.75 as fair to good, and < 0.40 as poor. A normal, two-tailed *p*-value of < 0.05 was considered statistically significant. Statistical analysis was performed using SPSS V. 24.0 (IBM Corp., Armonk, NY).

## Results

### Demographics

Fifty-three eyes of 29 patients (16 men and 13 women) were included in the study. The average age of the patients was 51.4 ± 9.5 years, and the average diabetes duration was 9.2 ± 5.2 years. The mean best-corrected visual acuity (BCVA) was 0.42 ± 0.40 logarithm of the minimum angle of resolution (LogMAR). The mean intraocular pressure (IOP) was 16.3 ± 2.4 mmHg. All enrolled patients were diagnosed with type 2 DM. Twenty-eight (52.8%) eyes were treatment-naïve, and 25 (47.2%) had a history of treatment before imaging (15 eyes of 8 patients with anti-VEGF intravitreal therapies [IVT],and the anti-VEGF drugs was Conbercept, 8 eyes of 5 patients with panretinal photocoagulation [PRP], and both eyes of 1 patient with both therapies). Twenty-three eyes of patients had concomitant diabetic macular edema, and one eye of one patient was diagnosed with DR secondary to NVG with eye drop-controlled IOP. All of these eyes underwent wfSS-OCTA and FFA examinations. The baseline characteristics of these patients are summarized in Table 1.

**Table 1:** Baseline characteristics of study participants (*N* = 53 eyes in 29 patients)

Patient characteristics	N (%) or mean ± SD
Sex	
Female	13 (44.8%)
Male	16 (55.2%)
Age (years)	51.4 ± 9.5
Laterality	
Right eye	28 (52.8%)
Left eye	25 (47.2%)
LogMAR BCVA	0.42 ± 0.40
IOP (mmHg)	16.3 ± 2.4
Diabetes duration (years)	9.2 ± 5.2
Prior treatment	25 (47.2%)
DME	23 (43.4%)
NVG	1 (1.9%)

*LogMAR* logarithm of the minimum angle of resolution, *BCVA* best-corrected visual acuity, *IOP* intraocular pressure, *DME* diabetic macular edema, *NVG* neovascular glaucoma

### Comparison between wfSS-OCTA VAMIs and resembling FFA images

#### Detection rate of PDR

In 29 (54.7%) eyes, NV was detected by OCTA, and in 25 (47.2%) eyes, NV was detected by FFA (Table 2). In all 25 (47.2%) eyes in which NV was detected by FFA, the finding was also confirmed by OCTA. In 24 (45.3%) eyes, absence of NV was confirmed by both FFA and OCTA examination. In four (7.5%) eyes with NV identified on OCTA, FFA did not confirm NV, and these patients had all received anti-VEGF IVT within the past 1 month. The wfSS-OCTA VAMIs were comparable to resembling FFA images in discovering NV (*p* = 0.125). The level of agreement was excellent (κ = 0.850, *p* < 0.001).

**Table 2:** Detection rate of neovascularization and neovascularization of the disc in diabetic retinopathy patients (*N* = 53 eyes in 29 patients)

	OCTA	FFA	*P* value	Cohen’s Kappa
NV (eye/N)	29/53 (54.7%)	25/53 (47.2%)	0.125	0.850, *p* < 0.001
NVD (eye/N)	11/53 (20.8%)	9/53 (17.0%)	0.625	0.754, *p* < 0.001

*NV* neovascularization, *NVD* neovascularization of the disc, *DR* diabetic retinopathy, *OCTA* optical coherence tomography angiography, *FFA* fluorescein fundus angiography

Using resembling FFA as the gold standard, the sensitivity for detection of NV with OCTA VAMIs was 100.0% (95% confidence interval [CI]: 83.41%, 100.00%), the specificity was 85.7% (95% CI: 66.44%, 95.32%), the positive-predictive value was 86.2% (95% CI: 67.43%, 95.49%), the negative-predictive value was 100.0% (95% CI: 82.83%, 100.00%), and the accuracy was 92.4%.

#### Detection rate of NVD

In 11 (20.8%) eyes, NVD was detected using OCTA, and in nine (17.2%) eyes, NVD was detected using FFA, of which NVD in eight (15.1%) eyes was confirmed by both FFA and OCTA examination (Table 2). No NVD was found in 41 (77.4%) eyes by both FFA and OCTA examinations. In one eye (1.9%), NVD was detected with FFA, but not with OCTA, due to the location of the NVD on the surface of the ILM, which resulted in segmentation errors. In three (6.0%) eyes, NVD was detected by OCTA but were missed by FFA. These patients had received anti-VEGF IVT within the past 1 month. WfSS-OCTA VAMIs were comparable to resembling FFA images in terms of the detection rate of NVD (*p* = 0.625). The level of agreement was excellent (κ = 0.754, *p* < 0.001, Table 2). When the sum of the discrepancy values between FFA and OCTA was < 25, the exact McNemar’s test was used.

#### Numbers of NVEs

**Fig. 3:**
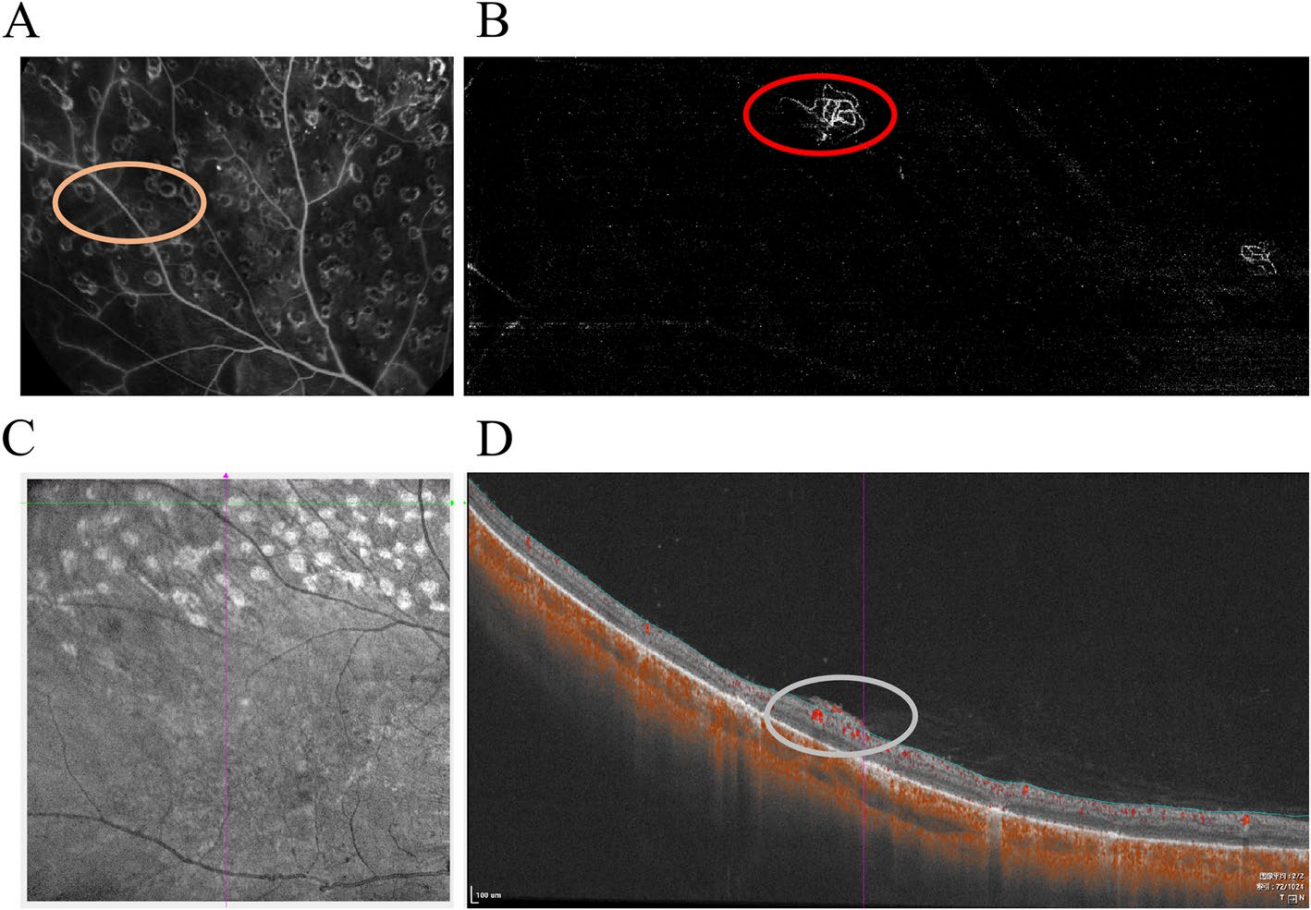
A 39-year-old man with a history of panretinal photocoagulation was treated with anti VEGF IVT 16 days ago. **A** Compared with Fig. 1C (red circle), there wasn’t NVE-like leakage in FFA (orange ellipse). **B** Supratemporal VAMI: Compared with Fig. 1A (red circle), abnormal vessels blood flow can be seen has pruned vascular loops of filamentous new vessels (red ellipse). **C**, **D** A confocal scanning laser ophthalmoscopy (cSLO) image and horizontal B-scan image show layer segmentation of the corresponding OCTA VAMI shown on (**B**). Compared with Fig. 1B (red circle), the NVE shows reduced hyper-reflected blood signal next to the anterior surface of the retina (gray ellipse)

The number of NVEs detected using OCTA and FFA differed significantly (Z = -2.265, *p* = 0.024 < 0.05). Ninety and 72 NVEs were identified using OCTA and FFA, respectively (Table 3). Four NVEs were identified using FFA but were not detected when OCTA was used. In two of these four NVEs, this was due to abnormality of small new vessels immediately above the retinal surface, which was not found in automatic computer segmentation. The other two NVEs were located at the edge of the VAMIs and were lost. Twenty-two NVEs were found in 11 eyes by using OCTA but lost in FFA. Five of 11 eyes were missed due to fluorescence leakage of small NVEs that coincided with a hyper-fluorescence background of retinal photocoagulation scars and retinal vessels with FFA. Interestingly, six of the 11 eyes underwent imaging < 1 month after receiving anti-VEGF IVT. The missed NVEs of the 6 treated eyes observed as faint or unconspicuous leakage in FFA. The morphology of these NVEs tend to be large-trunk vessels, pruned vascular loops, decreased branches in OCTA VAMI, and reduced abnormal hyper-reflected blood flow signals next to the anterior surface of the retina in B-scan (Fig. 3). If the seven eyes of the four patients who received anti-VEGF IVT within less than 3 months were excluded, OCTA was comparable to FFA in discovering the number of NVEs (Z = -1.273, *p* = 0.203). The level of agreement was excellent (ICC = 0.941, *p* < 0.001); 79 NVEs were detected using OCTA, and 71 NVEs were detected using FFA (Table 3).

**Table 3:** The number of neovascularization elsewhere (NVE)

	OCTA	FFA	*P* value	ICC
NVEs A (53 eyes in 29 patients)	90	72	0.024	0.912, *p* < 0.001
NVEs B (46 eyes in 25 patients)	79	71	0.203	0.941, *p* < 0.001

NVEs A: the number of NVEs in 53 eyes with DR

NVEs B: the number of NVEs in 46 eyes with DR which were excluded the images of the seven eyes from four patients who received anti-VEGF IVT less than 3 months

### Comparison of merged FFA vs. resembling FFA

Use of resembling FFA images was comparable to use of merged FFA images in diagnosing PDR (*p* = 0.500). The level of agreement was excellent (κ = 0.925, *p* < 0.001). Twenty-seven (50.1%) eyes were diagnosed with PDR using merged FFA, and 25 (47.2%) eyes were diagnosed with PDR using resembling FFA (Table 4). Two (3.8%) eyes were diagnosed with PDR using merged FFA images, but not with resembling FFA images because the NV of these patients was located outside the coverage area of the resembling FFA images. A total of 104 NVEs in 24 eyes and 72 NVEs in 21 eyes were discovered using merged FFA images and resembling FFA images, respectively (Table 4). The number of NVEs identified using merged FFA and resembling FFA images differed statistically significantly (Z = 3.461, *p* = 0.001). The level of agreement was excellent (ICC = 0.866, *p* < 0.001). Of the 104 NVEs in 53 eyes, 32 NVEs were distributed outside the resembling FFA image; however, in only three eyes, all the NVEs were located outside the resembling FFA images, and in one of these three eyes, an NVD was found within the resembling FFA image. The diagnosis of PDR was affected in only two eyes, in which all NVs were outside the coverage area of the resembling FFA image.

**Table 4:** Comparison of merged FFA vs. resembling FFA (*N* = 53 eyes in 29 patients)

	**Resembling FFA**	**Merged FFA**	*P* value	
**NV** (eye/N)	25/53	27/53	0.500	κ = 0.925, *p* < 0.001
**NVD** (eye/N)	9/53	9/53		
**NVE**(eye/N, number)	21/53, 72 NVEs	24/53, 104 NVEs	0.001	ICC = 0.866, *p* < 0.001

*NV* neovascularization, *NVD* neovascularization of the disc, *NVE* neovascularization elsewhere, *FFA* fluorescein fundus angiography, *ICC* intraclass correlation coefficient

Using merged FFA as the gold standard, the sensitivity for detection of NVs with resembling FFA images was 92.6% (95% CI: 74.25%, 98.71%), the specificity was 100% (95% CI: 83.98%, 100%), the positive-predictive value was 100.0% (95% CI: 83.42%, 100.00%), the negative-predictive value was 92.86% (95% CI: 75.04%, 98.75%), and the accuracy was 96.2%.

## Discussion

OCTA is a useful tool for identifying the origins and morphological patterns of NV in PDR [17, 24]. Previous studies on eyes with DR reported that using OCTA with different scanning modes was useful in detecting DR lesions [16, 20, 22, 23, 25–27]. The detection rate of NV with OCTA was the same as that with resembling FFA [25, 28]. In this prospective study, five fixation points with the 12 × 12 mm scan protocol of wfSS-OCTA (VG200) were used to expand the retinal scan area up to 17.5 × 23.5 mm to compare with that of resembling FFA images for NV identification in patients with severe NPDR or PDR. The results showed that wfSS-OCTA was comparable to FFA in identifying NVs and detecting NVDs and NVEs. The level of agreement was excellent (κ = 0.850, κ = 0.754, ICC = 0.941). A previous prospective study also showed that 15 × 15-mm OCTA (PLEX Elite 9000) was comparable to ultra-widefield (UWF) FFA in terms of the detection rate of NV, but was inferior in terms of the number of NVE identified [9]. Using FFA as the gold standard, the sensitivity for detection of NV in PDR by OCTA was 96.55%, and the specificity was 94.74%, which was similar to our findings. Another study reported that using 12 × 12-mm wfSS-OCTA (PLEX Elite 9000) with five fixation points was comparable to UWF-FA in terms of diagnostic accuracy for treatment-naïve patients with active PDR [23]. A retrospective study showed that the efficacy of SS-OCTA VRI slab images of 15 × 15-mm wfSS-OCTA for detecting NV in PDR eyes was comparable to that of FFA [16]. Hirano T et al. reported that wfSS-OCTA (23 × 20 mm) using Xephilio OCT-S1 identifed nearly all NVs in eyes with PDR [29]. Wide-angle OCTA seems to be a clinically useful tool for detecting non-perfusion areas or NVs [25, 28]. These results are consistent with our own findings. Taken together, the evidence indicates that wfSS-OCTA is as useful as resembling FFA for detecting NV and for identifying NVD and NVE.

NVEs are more likely to be located in the posterior pole than in the far periphery [30]. A study demonstrated that on UWF FFA, 63% of NVEs were located in the temporal quadrants with 93% of them being detected on WF-OCTA [31]. Feman et al. (1998) reported that NV was found against a background of DR in 282 eyes of 3,121 patients after follow-up over a 1-year period [32]. The mean distance from the optic nerve to the first site of NVE was 6.0 mm. Russell et al. (2019) reported that NV was detected in 98.3% of 651 PDR eyes using wfSS-OCTA, with UWF-FFA as reference [33]. NV mainly occurs in the posterior pole, rather than in the retinal periphery, for the following reasons: first, it may be that the peripheral areas of greatest non-perfusion are severely injured and thus incapable of mounting a proliferative response with VEGF stimulation. Thus, NVE may arise in these healthier areas posteriorly that are capable of responding to the rising levels of VEGF [30]. Second, in regions where there is complete vitreous separation, there may not be a favorable scaffold for NV to develop and organize, and thus these areas may be relatively resistant to NV development. Conversely, areas with a firm vitreous attachment (e.g., at the disc or along the vascular arcades) may be predisposed to proliferation [34]. Third, as an angiogenic inhibitor, pigment epithelium-derived factor is mainly released by the peripheral retinal pigment epithelial (RPE) cells, rather than by the macular RPE cells [35]. The field of view in merged FFA images is wider than that in resembling FFA images (similar to wfSS-OCTA VAMIs). In our study, NVs were found in 25 of 27 (92.6%) eyes with PDR when using resembling FFA, as compared with merged FFA. Therefore, a few instances of NV were detected in merged FFA images, but not in resembling FFA images (Fig. 2E). Our results suggested that wfSS-OCTA may be sufficient to identify the majority of NV instances in the mid-periphery of the retina in patients with PDR [14]. However, we have utilized Heidelberg machine for FFA which may be inferior to detect peripheral NVs than UWF FFA, and the colour fundus (CF) photography may prove to be even inferior than that of FFA. Some of the peripheral lesions that were not picked up on wfSS-OCTA and FFA, UWF FFA with UWF CF may be an option. So further studies between wfSS-OCTA with UWF-FFA may be needed. Interestingly, four eyes with NVs and three eyes with NVDs were identified on wfSS-OCTA VAMIs, but not on FFA images. These eyes had a history of previous treatment with anti-VEGF IVT. There were differences between wfSS-OCTA VAMIs and resembling FFA images in terms of discovering the number of NVEs in our study. However, when we excluded patients who underwent imaging within 3 months of anti-VEGF IVT, wfSS-OCTA VAMIs were comparable to FFA images in terms of discovering the number of NVEs, with an excellent level of agreement (ICC = 0.941, *p* < 0.001).

A pilot study obtained similar results in patients with PDR who underwent anti-VEGF IVT [36]; the structure of the NV was maintained while its flow density and angio-area regressed after anti-VEGF IVT. Previous observations showed that regressed NV remained as a truncated vessel loop, showing no leakage on FFA [18]. NV, a characteristic of PDR, is induced by the high expression of VEGF [37]. VEGF increases vascular permeability, resulting in the leakage of proteins and other molecules from the blood vessels [38] and promoting NV phenomena [39]. NV is characterized by abnormal vessels showing definite fluorescein dye leakage in FFA. Therefore, after treatment with anti-VEGF agent, it is difficult to identify the regressed NV, which has reduced fluorescein leakage, even though NV can be identified as a vascular-like blood flow signal on wfSS-OCTA VAMIs. The flow signal represents the movement of erythrocytes in wfSS-OCTA that is repeated over a short time period. As long as erythrocyte movement occurs within the abnormal vessels in the vitreous cavity, the signal of vascular-like blood flow can be detected in wfSS-OCTA VAMIs, even if the NV flow signal in the OCTA images may be decreased after anti-VEGF injection [28, 36, 40]. The clinical grade of DR might be underestimated with FFA because of NV regression or disappearance after anti-VEGF IVT, which has a potential risk for leading to VH after a period of time. Non-invasive OCTA may detect tiny NV in patients after anti-VEGF therapy, who can then be diagnosed with PDR. The development of the disease can be estimated, the prognosis judged, and a reasonable follow-up strategy developed. However, it is important to notice that after anti-VEGF injection, not all the NVs were without leakage on FFA. Some of them still can be picked up on FFA as diffuse leaks in early stage. Hence, the use of FFA cannot be completely ignored. A large-scale prospective controlled study is needed to clarify the changes in NV further among patients with PDR on OCTA and FFA after anti-VEGF IVT.

In this study, NV was found in five eyes by OCTA, but not by FFA examination. Two eyes showed a large amount of fluorescence leakage, which obscured the features of NVEs, and three eyes had fluorescence leakage at small NVEs, coinciding with a hyper-fluorescence background of retinal photocoagulation scars. With no impact on fluorescein leakage, wfSS-OCTA VAMI may be more sensitive than FFA in detecting NV in patients with poor cooperation, diffuse leakage of abnormal vessels, or minimal dye leakage of NV. Therefore, with 3D flow imaging, even small-sized NV located closely in front of strong fluorescent areas, such as laser scars or retinal blood vessels in FFA, can be easily found on VAMIs, when excluding the blood flow behind the ILM. In addition, the single, auto-stitched wide-angle VAMI (17.5 × 23.5 mm) makes the white NV clear on the black background, which is convenient for the graders to find out NV and reduces the time spent in detecting NV in B scan frame by frame. VAMIs may be useful to detect and monitor NV in clinical practice. Thus, detecting and following-up changes in NV with OCTA VAMI is more advantageous than doing so with FFA for DR patients with a history of previous treatment with anti-VEGF IVT or PRP.

NVs from the retinal and optic nerve head that grow into the vitreous cavity and show marked leakage visualized in FFA are the diagnostic hallmarks of PDR [15, 16]. A VRI slab (in the PLEX Elite 9000) showing a high reflection of blood flow from the automatically detected retinal surface or from 10 μm above the ILM to 200 µm or 300 µm above the ILM/vitreous cavity was proven to be useful in the detection of NV in patients with DR [9, 16, 18, 20, 21, 23]. In our study, VAMI (in the VG 200) revealed a high reflection of blood flow from 5 μm above the ILM (automatically detected) to the top vitreous cavity in the B-scan, which is also useful in the display of NV in patients with DR. Although differing in scope definition, the ability to diagnose NV between VAI slabs and VAMIs were the same. However, the detection of IRMA has multiple uncertainties in OCTA RAMI. In this study, NVD in one (1.9%) eye and NVEs in two (3.8%) eyes were identified by FFA but not by wfSS-OCTA VAMI. The missed diagnosis NVD and NVE were all small and adjacent to the surface of the ILM, resulting in auto-segmentation errors, and were misdiagnosis of IRMA in OCTA RAMI by the graders. Auto-segmentation errors of the retinal layers have been reported as a major artifact source in OCT images of healthy and DR eyes [41], and can be corrected by manual segmentation [42]. Indeed, in a previous study, after manually correcting segmentation, the sensitivity of VRI slab images for detecting neovascularization increased from 73 to 84% [16]. Therefore, attention should be paid to misdiagnosis caused by auto-segmentation errors.

As the eye is a sphere, the resembled eyeball is nearly flat when the scan coverage is small (3 × 3 mm), and the whole retina can be contained in the scan volume. With the expansion of coverage (17.5 × 23.5 mm), the resembled eyeball is nearly hemispherical. If we focus on the central part of the retina, the surrounding parts will bulge out of the scan volume. In our study, more NVEs located at the edge of the scan areas of RAMI were found in resembling FFA images than in VAMIs in two eyes. Usually, VAMI and RAMI have the same scan area. Sometimes, the surrounding parts of VAMI are cut off because they bulge out of the vitreous scan volume, resulting in a smaller scan area than that of RAMI. OCTA could only display the image of the central part of the posterior pole retina in patients with severe myopia, which restricted the use of OCTA in the independent diagnosis of PDR in DR patients with severe myopia. Therefore, the challenge for wfSS-OCTA is not only to expand the scan width but also to increase the scan depth accordingly.

The limitations of the current study included the small sample size and that naïve and treated patients were not analyzed separately. To validate our conclusions, it will be necessary to perform a large-scale randomized and multicenter clinical trial.

In conclusion, wfSS-OCTA is an independent alternative to FFA for diagnosing PDR, detecting NVD, and identifying individual NVE non-invasively, particularly for patients with PDR who have undergone previous treatment.

## Data Availability

The datasets used and/or analysed during the current study are available from the corresponding author on reasonable request.

## Abbreviations

DM: Diabetes mellitus
VAMI: Vitreous angiomosaic image
wfSS-OCTA: Widefield swept-source optical coherence tomography angiography
NV: Neovascularization
ILM: Internal limiting membrane
RAMI: Retinal angiomosaic image
IRMA: Intraretinal microvascular abnormalities
FFA: Fluorescein fundus angiography
NVD: Neovascularization of the disc
NVE: Neovascularization elsewhere
OCT: Optical coherence tomography
DR: Diabetic retinopathy
NPDR: Non-proliferative diabetic retinopathy
PDR: Proliferative diabetic retinopathy
VEGF: Vascular endothelial growth factor
VH: Vitreous hemorrhage
NVG: Neovascular glaucoma
2D: Two-dimensional
MA: Microaneurysm
3D: Three-dimensional
VRI: Vitreoretinal interface
AMI: Angiomosaic image
SD: Standard deviations
ICC: Intraclass correlation coefficient
BCVA: Best-corrected visual acuity
LogMAR: Logarithm of the minimum angle of resolution
IOP: Intraocular pressure
VEGF: Vascular endothelial growth factor
IVT: Intravitreal therapies
PRP: Panretinal photocoagulation
DME: Diabetic macular edema
CI: Confidence interval
